# A New Method to Detect Buffalo Mastitis Using Udder Ultrasonography Based on Deep Learning Network

**DOI:** 10.3390/ani14050707

**Published:** 2024-02-23

**Authors:** Xinxin Zhang, Yuan Li, Yiping Zhang, Zhiqiu Yao, Wenna Zou, Pei Nie, Liguo Yang

**Affiliations:** 1 National Center for International Research on Animal Genetics, Breeding and Reproduction (NCIRAGBR), Ministry of Science and Technology of the People’s Republic of China, Huazhong Agricultural University, Wuhan 430070, China; 1144795936@webmail.hzau.edu.cn (X.Z.); liyuan@webmail.hzau.edu.cn (Y.L.); zhangyiping1998@webmail.hzau.edu.cn (Y.Z.); zhiqiuyao@webmail.hzau.edu.cn (Z.Y.); qiuyi@webmail.hzau.edu.cn (W.Z.); 2 Key Laboratory of Animal Genetics, Breeding and Reproduction, Ministry of Education, College of Animal Science and Technology, Huazhong Agricultural University, Wuhan 430070, China; 3 College of Veterinary Medicine, Hunan Agricultural University, Changsha 410128, China; niepei20190602@163.com

**Keywords:** PolyLoss, convolutional block attention module, somatic cell count, quarter, mastitis

## Abstract

**Simple Summary:**

In this study, deep learning combined with udder ultrasonography of buffalo was used for the detection of mastitis for the first time, with the aim of establishing an accurate, rapid, and inexpensive method to detect buffalo mastitis instead of routine laboratory examination. This method provides a basis for mastitis detection in buffaloes mostly raised by small farmers and has the opportunity to be used in a variety of dairy animals in the future.

**Abstract:**

Mastitis is one of the most predominant diseases with a negative impact on ranch products worldwide. It reduces milk production, damages milk quality, increases treatment costs, and even leads to the premature elimination of animals. In addition, failure to take effective measures in time will lead to widespread disease. The key to reducing the losses caused by mastitis lies in the early detection of the disease. The application of deep learning with powerful feature extraction capability in the medical field is receiving increasing attention. The main purpose of this study was to establish a deep learning network for buffalo quarter-level mastitis detection based on 3054 ultrasound images of udders from 271 buffaloes. Two data sets were generated with thresholds of somatic cell count (SCC) set as 2 × 10^5^ cells/mL and 4 × 10^5^ cells/mL, respectively. The udders with SCCs less than the threshold value were defined as healthy udders, and otherwise as mastitis-stricken udders. A total of 3054 udder ultrasound images were randomly divided into a training set (70%), a validation set (15%), and a test set (15%). We used the EfficientNet_b3 model with powerful learning capabilities in combination with the convolutional block attention module (CBAM) to train the mastitis detection model. To solve the problem of sample category imbalance, the PolyLoss module was used as the loss function. The training set and validation set were used to develop the mastitis detection model, and the test set was used to evaluate the network’s performance. The results showed that, when the SCC threshold was 2 × 10^5^ cells/mL, our established network exhibited an accuracy of 70.02%, a specificity of 77.93%, a sensitivity of 63.11%, and an area under the receiver operating characteristics curve (AUC) of 0.77 on the test set. The classification effect of the model was better when the SCC threshold was 4 × 10^5^ cells/mL than when the SCC threshold was 2 × 10^5^ cells/mL. Therefore, when SCC ≥ 4 × 10^5^ cells/mL was defined as mastitis, our established deep neural network was determined as the most suitable model for farm on-site mastitis detection, and this network model exhibited an accuracy of 75.93%, a specificity of 80.23%, a sensitivity of 70.35%, and AUC 0.83 on the test set. This study established a 1/4 level mastitis detection model which provides a theoretical basis for mastitis detection in buffaloes mostly raised by small farmers lacking mastitis diagnostic conditions in developing countries.

## 1. Introduction

The buffalo is a domestic animal with strong adaptability, superior production performance, good heat tolerance, and high roughage utilization efficiency [1]. Compared to cow’s milk, buffalo milk is rich in fat, lactose, protein (especially casein), and minerals (such as magnesium, calcium, and inorganic phosphate) [2]. In addition, buffalo milk production has a significant impact on global milk production [3]. The buffalo industry plays a vital role in economic development, rural livelihood, and poverty alleviation, and it can also meet the population’s rapidly growing demand for animal protein to some degree [4]. Despite the valuable contributions of buffaloes to agriculture and human well-being, the buffalo industry faces emerging challenges [5].

In veterinary medicine, mastitis is considered as one of the most common and economically important diseases that adversely affects lactating dairy herds worldwide [6]. With the growing popularity of buffalo milk and its dairy products, a higher buffalo milk yield is pursued, which has resulted in a gradual increase in the incidence of mastitis in buffaloes. However, there is a lack of relevant research on buffalo mastitis. Mastitis usually results in inflammation of one or more quarters of the mammary glands, which often affects not only a single animal, but also the entire herd, or at least multiple individuals within a herd [7]. Rapid, accurate, and economical mastitis diagnosis methods are very important in order to reduce the losses caused by mastitis. Traditional mastitis detection methods include somatic cell count (SCC), inflammation indication, detection of biomarkers related to disease onset, and identification of pathogenic microorganisms, but these methods have limitations [8]. Somatic cell count is a useful indicator for detecting mastitis, but it is a long and time-consuming procedure, and the dairy industry needs fast, sensitive, specific, and reliable detection methods [9].

Udder ultrasound detection is an easy-to-operate, non-invasive technique that allows for the visualization and health assessment of teats, teat canal, gland cisterns, and mammary gland parenchyma, which contributes to decision making about culling or treating diseased animals [10]. Mastitis caused by an udder infection with staphylococcus aureus and the corresponding pathological changes in the udder can be visualized by ultrasound [11]. Due to the complexity of medical images, the scarcity of medical experts, the subjectivity of diagnosis, and other factors, it is very difficult to determine udder health status through ultrasound images. The rapid advancement of advanced computing and imaging technologies has led to the emergence of a novel research domain known as computer-aided diagnosis systems, which are utilized across diverse biomedical applications [12]. Among them, the emergence of deep learning provides accurate, convenient, and efficient solutions for medical image-based diagnosis.

Over the past decade, machine learning and deep learning have become sub-fields of artificial intelligence, exhibiting excellent performance in healthcare industry applications since they can reduce costs while increasing efficiency [13]. It may take clinicians 30–60 min to evaluate all animal information and make a herd mastitis diagnosis, but it may take machine learning algorithm only a few seconds to complete the same tasks, and this increases the detection rate and gives clinicians more time to focus on implementing mastitis control strategies [14]. Deep neural networks have several obvious advantages, since they can learn high-level abstract features directly from raw images or data. In addition, deep neural networks can directly output individual prediction labels for each input image to classify the targets of interest [15]. In recent years, mastitis deep learning detection networks based on milking parameters or various physiological manifestations of animals have become a new tool with promising applications [16,17,18].

Water buffalo holds significant economic importance, particularly for small-scale producers in developing countries [19]. The backward economy and breeding technology have led to a serious lack of conditions and technologies for detecting mastitis, making it impossible to ensure the accuracy and timeliness of mastitis detection, thus causing huge economic losses and hindering the development of the buffalo dairy industry. The scientific intelligent detection method of mastitis established by employing deep learning networks to extract ultrasonic imaging features of healthy and mastitis-stricken udders is urgently needed for the development of the buffalo industry, which currently uses backward breeding technology. To the best of our knowledge, to date, there has been no deep learning model for mastitis detection based on ultrasound images of the udder. This study provides a methodological basis for on-site quarter-level mastitis detection on buffalo farms and promotes the healthy development of the buffalo dairy industry.

## 2. Methods and Materials

### 2.1. Data

The data were collected on the buffalo farms of Hubei Jinniu Animal Husbandry Co., Ltd. and Guangxi Buffalo Research Institute in China from 2020 to 2022. The buffalo udder ultrasound imaging was performed by a trained researcher using a portable ultrasound device equipped with a 3–7.5 MHz linear array transducer (WED-3000-v, equipped with LNA/6.5 MHz rectal probe, Shenzhen Well; D Medical Electronics Co., Ltd., Guangdong, China), with the frequency set at 5–7.5 MHz. The procedures to obtain udder ultrasound images were as follows. If hair was present in the area where the ultrasound probe was positioned on the udder and could potentially interfere with the effectiveness of ultrasound measurements, it was removed. Ultrasound gel was applied to the probe to enhance contact with the animal’s udder skin. To ensure diversity of the ultrasound images, we conducted scans under different conditions. The collection of identical udder quarter ultrasound images occurred at various time points (before and after milking in the morning or afternoon) and on different scanning planes (coronal and sagittal planes). Additionally, measurements of the same udder were spaced 1–4 months apart to obtain ultrasound images of the same udder quarters at different lactation stages and with different health statuses.

The resolution of the image generated by ultrasound scanning was 800 × 553 pixels, and the gray value range was 0–255. All images were stored in BMP format in a USB flash drive through the B-ultrasound machine and transmitted to the computer for image processing. The milk samples of 1/4 udders were collected and placed in a 50 mL sterile vial containing preservatives. The milk samples were sent to the Hubei Center of Dairy Herd Improvement for SCC detection with a milk composition detector (CombiFoss FT+ FOSS Analytical, Hillerød, Denmark).

### 2.2. Establishment of a Deep Learning Network

#### 2.2.1. Data Set Composition

The SCC cutoff value for mastitis definition may be different in different regions, and 2 × 10^5^ cells/mL SCC is the most commonly used cutoff value for dairy cows [20]. In most developed dairy industries, such as the countries in European Union, define the SCC cutoff value of buffalo milk as 4 × 10^5^ cells/mL [21]. In this study, two data sets were generated with SCC threshold set as 2 × 10^5^ cells/mL or 4 × 10^5^ cells/mL, respectively. The udders with SCC ≥ the set threshold were defined as mastitis-stricken udders, and those with SCC < the threshold as healthy udders. The compositions of the two data sets are shown in Table 1.

#### 2.2.2. Establishment of a Mastitis Detection Network Based on Udder Ultrasound Images

The EfficientNets [22], as a network series, use a simple and efficient composite coefficient to achieve unified scaling of all dimensions of depth, width, and resolution. The EfficientNet_b7 model achieves state-of-the-art accuracy of 84.4% top-1/97.1% top-5 on ImageNet while outperforming the previous best ConvNet by reducing the size by 8.4 times and increasing the speed by 6.1 times. The convolutional block attention module (CBAM) was proposed by Woo et al. [23], including channel and spatial attention modules, which are independent and complementary in their focuses. The channel attention module focuses on what is meaningful in the input image, unlike channel attention, spatial attention attends to where the informative parts are located. PolyLoss is a simple framework that designs the loss function as a linear combination of polynomial functions, and this framework is inspired by the Taylor expansion of cross-entropy loss (1) and focal loss (2). PolyLoss outperformed cross-entropy loss and focal loss on tasks such as 2D image classification, target detection, and instance segmentation. In addition, PolyLoss has been proven to be beneficial for solving the imbalance of sample categories to a certain extent [24].

(1) $$L_{CE}=-\log\left(P_{t}\right)=\overset{\infty }{\underset{j=1}{\sum }}1/j{\left(1-p_{t}\right)}^{j}=\left(1-P_{t}\right)+1/2{\left(1-p_{t}\right)}^{2}\ldots$$

(2) $$L_{FL}=-{\left(1-P_{t}\right)}^{\gamma }\log\left(P_{t}\right)=\sum_{j=1}^{\infty }1/j{\left(1-p_{t}\right)}^{j+\gamma }={\left(1-P_{t}\right)}^{1+\gamma }\;+\;1/2{\left(1-p_{t}\right)}^{2+\gamma }\ldots$$

where $P_{t}$ is the predicted probability of the target ground-truth class by the model.

**Figure 1:**
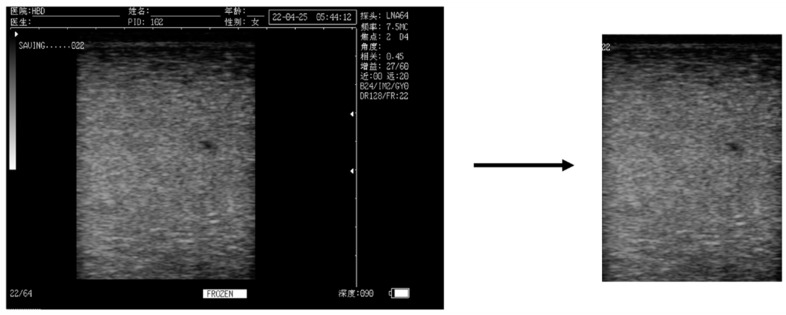
Preprocessing of udder ultrasound images. Note: The Chinese information in the udder ultrasound image pertains to the parameter settings during the process of capturing this photograph.

To avoid the interference of invalid pixels, after preprocessing, the ultrasound images of the udder were intercepted from the images with the complex information background (Figure 1). The two data sets were randomly divided into a 70% training set (2140 images), a 15% validation set (457 images), and a 15% test set (457 images), respectively. The training set and validation set were used for network development, and the test set was used for network performance evaluation. In order to prevent overfitting caused by excessive model parameters, and to avoid a model with an insufficient simple learning ability, we used the EfficientNet_b3 network and added one CBAMs before its last fully connected layer for mastitis detection model training. Polyloss was used as the loss function during the training process. In order to improve the generalization ability of the model, we used random horizontal flip, random vertical flip, random rotation, and others for data enhancement during each training session. The epoch was set as 100, the batch size as 32, and the starting learning rate as 0.0001. The cosine function was used to adjust the learning rate during the training process.

### 2.3. Model Performance Evaluation

To evaluate the mastitis detection performance of the network, we examined five commonly used classification model performance evaluation indicators, including accuracy (3), sensitivity (4), specificity (5), F1-score (6), and the area under the receiver operating characteristic curve (AUC), by comparing the model prediction results and the SCC detection results. These indicators were calculated according to the following formulas.

(3) $$\mathrm{Accuracy}=\frac{\mathrm{TP}+\mathrm{TN}}{\mathrm{TP}+\mathrm{FP}+\mathrm{TN}+\mathrm{FN}}$$

(4) $$\mathrm{Sensitivity}=\frac{\mathrm{TP}}{\mathrm{TP}+\mathrm{FN}}$$

(5) $$\mathrm{Specificity}=\frac{\mathrm{TN}}{\mathrm{TN}+\mathrm{FP}}$$

(6) $$F1-\mathrm{Score}=\frac{2\mathrm{TP}}{2\mathrm{TP}+\mathrm{FP}+\mathrm{FN}}$$

where true positive (TP) refers to the number of mastitis samples classified correctly; true negative (TN) indicates the number of healthy samples classified correctly; false positive (FP) represents the number of healthy samples falsely classified as mastitis; and false negative (FN) refers to the number of mastitis samples falsely classified as healthy.

## 3. Results and Discussion

Early detection of mastitis can prevent further deterioration of udder health, minimize tissue damage, and improve treatment effectiveness [17]. In this study, a total of 3054 udder ultrasound images were obtained, involving 271 buffaloes. The EfficientNet_b3 network was used as the baseline, and we established a network by combining CBAM and PolyLoss for mastitis detection model training. The performances of these networks are shown in Table 2. When SCC ≥ 2 × 10^5^ cells/mL was defined as mastitis, our established model exhibited an accuracy of 70.02%, a specificity of 77.93%, a sensitivity of 63.11%, and an F1-score of 69.21% on the test set, whereas when SCC ≥ 4 × 10^5^ cells/mL was defined as mastitis, the overall classification performance of the model was better, with an accuracy of 75.93%, a specificity of 80.23%, a sensitivity of 70.35%, and an F1-score of 71.79%.

Clinical diagnosis of diseased dairy cows heavily relies on professional veterinarians, whether in large-scale or small-scale farming operations. However, in certain regions, the lack of highly skilled veterinarians compromises the accuracy and timeliness of diagnoses, resulting in substantial economic losses. Moreover, even experienced veterinarians may misdiagnose similar symptoms due to a lack of comprehensive knowledge or experience [25]. Computer-aided diagnostic systems in medicine can utilize diagnostic rules to simulate human experts’ systems of making diagnostic decisions. Additionally, these systems possess feedback mechanisms through which they can infer new knowledge from diverse data, thereby improving their diagnostic performance over time [26].

An accurate, automated mastitis diagnostic tool holds great potential to assist non-specialist clinical veterinarians in quickly making a herd-level diagnosis and taking appropriate measures in a timely manner to control a disease which has extreme adverse effects animal health, welfare, productivity, and antimicrobial drug application [14]. The applicability of a detection method for conventional diagnosis depends on several factors, including cost, sensitivity, specificity, time to produce results, and the feasibility of large-scale milk sampling [27]. Common mastitis detection methods such as pathogen identification causing intramammary infections, milk conductivity testing, and biomarker assessment may excel in specificity, sensitivity, and other performance aspects compared to our network. However, most of them suffer from drawbacks such as high cost, time consumption, and the difficulty of on-farm implementation in cattle fields. The management conditions in animal husbandry vary greatly. Therefore, the detection techniques for mastitis should be diversified to meet the needs of animals raised under different breeding management conditions.

To date, multiple cow mastitis detection models have been established through machine learning, but to our knowledge, a deep learning detection network for buffalo mastitis has not been reported yet. In the report by Ebrahimi et al. [16], various prediction systems were applied to a large milking parameters set to obtain the best mastitis prediction model. The results showed that deep learning and gradient-boosted trees were superior to other models. The deep learning cow mastitis diagnostic model based on infrared thermal imaging technology has been reported in many studies [18,28]. However, using this detection technology to determine udder health is susceptible to external factors, leading to inaccurate detection of mastitis [18]. Furthermore, a mastitis detection network has been established based on matrix-assisted laser desorption/ionization time-of-flight mass spectrometry [29], sensors [30], cytometric fingerprinting [31], and other technologies. In several studies, deep learning-based mastitis detection exhibited an accuracy of above 80% [16,32], but the equipment or big data required for mastitis detection are highly likely to be unavailable to buffalo farms. Additionally, most of these technologies cannot detect mastitis at the 1/4 level. Detecting mastitis at the quarter level is advantageous for formulating more rational animal management, treatment, and prognosis plans.

The human visual system can quickly and accurately find prominent areas in complex scenes. Inspired by this phenomenon, the attention mechanism was introduced into computer vision. The attention mechanism has become an effective method to select important information in order to obtain superior results [33], and it has achieved great success in multiple visual tasks such as object detection, image classification, video understanding, semantic segmentation, image generation, multimodal tasks, 3D vision, and self-supervised learning [34]. The traditional attention mechanism based on convolutional neural networks pays more attention to the information in the channel domain and ignores the information in the spatial domain. The convolutional block attention module will adaptively infer the attention map along the two independent dimensions of channel and space, which is superior to the attention mechanism that only focuses on channel information. CBAM has been widely used in medical images and shows excellent model performance [35,36]; thus, we decided to add CBAM to the training network to enhance the model’s ability to learn mastitis features.

**Figure 2:**
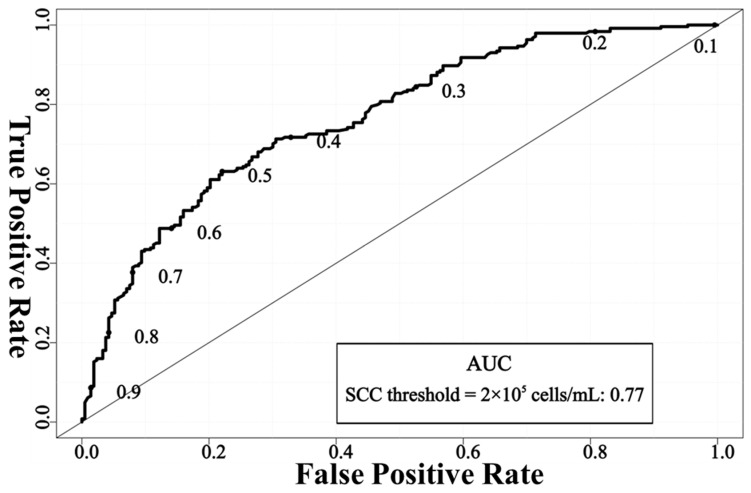
ROC curve of EfficientNet_b3 + CBAM + PolyLoss network framework for mastitis detection when SCC ≥ 2 × 10^5^ cells/mL was defined as mastitis. Note: SCC = somatic cell count; AUC = the area under the receiver operating characteristics curve.

**Figure 3:**
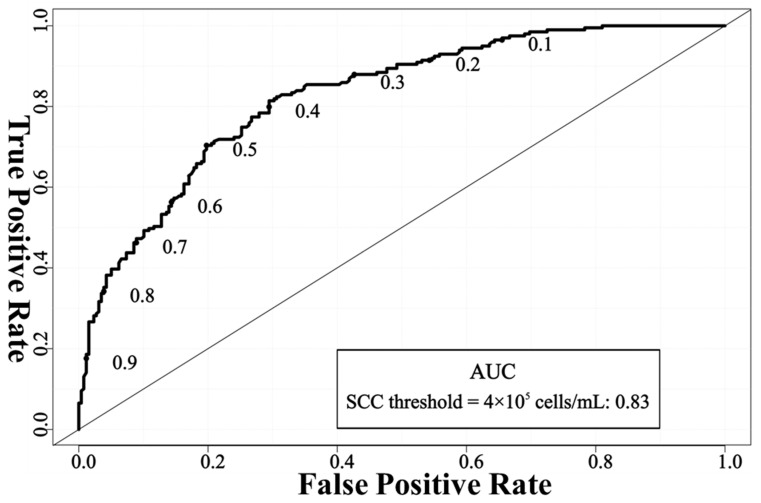
ROC curve of EfficientNet_b3 + CBAM + PolyLoss network framework for mastitis detection when SCC ≥ 4 × 10^5^ cells/mL was defined as mastitis. Note: SCC = somatic cell count; AUC = the area under the receiver operating characteristics curve.

The larger the AUC, the better the performance of the model in diagnosing mastitis. An AUC of 0.7–0.8 was defined as good, 0.8–0.9 as very good, and 0.9–1.0 as excellent [37]. Figure 2 (SCC threshold = 2 × 10^5^ cells/mL) and Figure 3 (SCC threshold = 4 × 10^5^ cells/mL) show the receiver operating characteristic curve (ROC) of the mastitis detection network used in this study. When SCC ≥ 2 × 10^5^ cells/mL was defined as mastitis, the AUC value of the mastitis detection network was 0.77, and when SCC ≥ 4 × 10^5^ cells/mL was defined as mastitis, the AUC value was 0.83. Judging from the AUC value, the detection performance of the model we established was a good model (at SCC threshold = 2 × 10^5^ cells/mL) and a very good model (at SCC threshold = 4 × 10^5^ cells/mL), respectively, indicating that the network is more capable of distinguishing between mastitis udders and healthy udders when SCC ≥ 4 × 10^5^ cells/mL is defined as mastitis.

**Figure 4:**
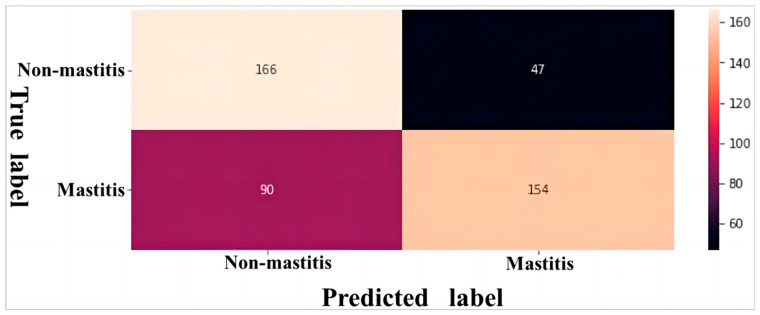
Confusion matrix of deep neural network for classifying buffalo udder ultrasound images on the test set when a somatic cell count ≥ 2 × 10^5^ cells/mL was defined as mastitis.

**Figure 5:**
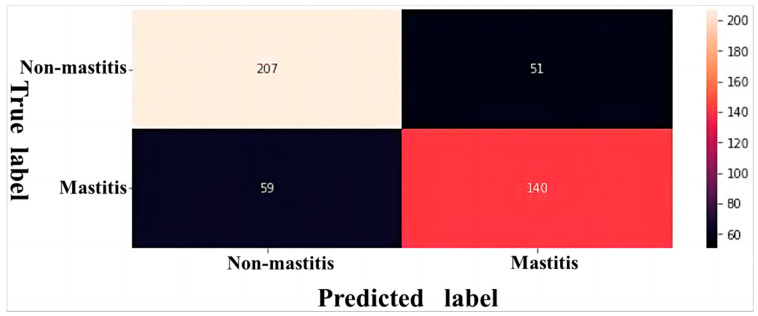
Confusion matrix of deep neural network for classifying buffalo udder ultrasound images on the test set when a somatic cell count ≥ 4 × 10^5^ cells/mL was defined as mastitis.

The classification performance of model was visualized through a confusion matrix. When SCC ≥ 2 × 10^5^ cells/mL was defined as mastitis, the network misclassified 90 mastitis samples (a total of 244 mastitis samples) into the healthy category and misclassified 47 healthy samples (a total of 213 healthy samples) into the mastitis category (Figure 4). When SCC ≥ 4 × 10^5^ cells/mL was defined as mastitis, the network misclassified 59 mastitis samples (a total of 199 mastitis samples) into the healthy category, but only misclassified 51 healthy samples (a total of 258 healthy samples) into the mastitis category (Figure 5). Both networks were more capable of identifying healthy udders than mastitis udders. However, the network’s classification was more accurate in identifying both mastitis-stricken and healthy udders at a SCC threshold = 4 × 10^5^ cells/mL than at a SCC threshold = 2 × 10^5^ cells/mL. After comprehensive evaluation, the deep neural network with SCC ≥ 4 × 10^5^ cells/mL defined as mastitis was determined as the most suitable for on-site buffalo mastitis detection in cattle farms.

Ultrasonography can present parameters such as mammary cistern and teat area, which can be used for assessing the udder health of lactating cows, and can reveal parenchymal damages such as mastitis, edema, hematoma, abscess, atrophy, and mammary fibrosis based on different echogenicities of various structures [38]. Ultrasound scanning of the mammary parenchyma at hour 72 after Staphylococcus infection showed a large range of hyperechoic areas, the lactiferous ducts became quite narrow, and the anechoic blood vessels could not be displayed [11]. Zhang et al. [39] used computer-assisted technology to quantify the texture of udder ultrasound images and found that there was a positive correlation between the pixel standard deviation of udder ultrasound texture and the SCC level, indicating that, as the mastitis became increasingly severe, the pathological manifestations corresponding to the ultrasound texture became more obvious. The report by Abdullah et al. [40] found that, as mastitis intensifies, the average numerical pixel values and pixel heterogeneity of the udder parenchyma echotexture increased. This explains our results that the detection performance of the model using SCC ≥ 4 × 10^5^ cells/mL as the threshold to classify mastitis was better than the model using SCC ≥ 2 × 10^5^ cells/mL.

Different cattle farms around the world choose different mastitis detection methods due to differences in geography, income, and herd size. Inexpensive detection technologies are especially important for buffalo farms in smallholder economies in developing countries. Ultrasound machines are the most ubiquitous reproductive tools in cattle farm management. The easy acquisition of udder ultrasound images (due to the simple measurement method and popularization of ultrasound equipment) and the powerful feature extraction capabilities of deep learning networks make it feasible to establish a buffalo mastitis detection method based on deep learning and udder ultrasonography, which will meet the urgent needs of cattle farms with backward breeding conditions. In this study, the optimal accuracy rate of the model in detecting mastitis was 75.93%, and our model could accurately locate the area where mastitis occurred at the 1/4 level, providing a basis for the formulation of subsequent treatment schemes and production management decisions.

## 4. Conclusions

This study used 4 × 10^5^ cells/mL and 2 × 10^5^ cells/mL SCC as thresholds, respectively, to train the 1/4 level buffalo mastitis detection network. The results showed that the network showed the optimal performance in terms of mastitis detection when SCC ≥ 4 × 10^5^ cells/mL was defined as mastitis. Compared with laboratory mastitis detection methods, which require expensive precision instruments and professional operators, the method established in this study was relatively less accurate, but it is still of great value to buffalo farms with relatively backward breeding technology. In the future, there is an opportunity to further expand the sample size of this study on cows or other dairy animals, which is very promising to improve the accuracy of the deep learning network for mastitis detection.

## Figures and Tables

**Table 1:** Number of udder ultrasound images in the two data sets of the mastitis group and the healthy group.

Data Set.	SCC Threshold	Healthy Group (*n*)	Mastitis Group (*n*)
1	2 × 10^5^ cells/mL	1424	1630
2	4 × 10^5^ cells/mL	1722	1332

Note: SCC = somatic cell count.

**Table 2:** Classification performance of EfficientNet_b3 + CBAM + PolyLoss network framework in buffalo mastitis detection with somatic cell count thresholds of 2 × 10^5^ cells/mL and 4 × 10^5^ cells/mL, respectively.

SCC Threshold	Accuracy (%)	Specificity (%)	Sensitivity (%)	F1-Score (%)
2 × 10^5^ cells/mL	70.02	77.93	63.11	69.21
4 × 10^5^ cells/mL	75.93	80.23	70.35	71.79

Note: SCC = somatic cell count; CBAM = convolutional block attention module.

## Data Availability

The data provided in this study can be requested from the corresponding author under reasonable circumstances. The data are not publicly available due to the privacy and confidentiality agreements as well as other restrictions.
